# MiR-92a promotes stem cell-like properties by activating Wnt/β-catenin signaling in colorectal cancer

**DOI:** 10.18632/oncotarget.21667

**Published:** 2017-10-09

**Authors:** Guang-Jun Zhang, Li-Fa Li, Guo-Dong Yang, Shu-Sen Xia, Rong Wang, Zheng-Wei Leng, Zuo-Liang Liu, Hong-Peng Tian, Yi He, Chang-Yuan Meng, Dai-Zhi Liu, Song-Lin Hou, Xue-Gui Tang, Tong Zhou

**Affiliations:** ^1^ The Second Department of Gastrointestinal Surgery, The Affiliated Hospital of North Sichuan Medical College, Nanchong, Sichuan, China; ^2^ Institute of Hepatobiliary, Pancreatic and Intestinal Disease, North Sichuan Medical College, Nanchong, Sichuan, China; ^3^ Department of Gastroenterology, North Sichuan Medical College, Nanchong, Sichuan, China; ^4^ Department of Gastrointestinal Surgery, The Affiliated Hospital of Zunyi Medical College, Zunyi, Guizhou, China; ^5^ Anorectal Department of Integrated Traditional Chinese and Western Medicine, North Sichuan Medical College, Nanchong, Sichuan, China

**Keywords:** colorectal cancer, miR-92a, stem cell, IL-6/STAT3, Wnt/β-catenin pathway

## Abstract

We previously reported the oncogenic function of miR-92a in colorectal cancer. This study identified that miR-92a was upregulated in chemoresistant colorectal cancer cells and tissues. Ectopic expression of miR-92a conferred resistance to 5-fluorouracil-induced apoptosis *in vitro*, while antagomiR-92a significantly enhanced chemosensitivity *in vivo*. Moreover, Overexpression of miR-92a promoted the tumor sphere formation and the expression of stem cell markers. MiR-92a overexpression also displayed higher tumourigenesis *in vivo*. Furthermore, we demonstrated that miR-92a upregulates the Wnt/β-catenin signaling activity via directly targeting KLF4, GSK3β and DKK3, which are multiple level negative regulators of the Wnt/β-catenin signaling cascade. In addition, our results indicate IL-6/STAT3 pathway increases miR-92a expression by directly targeting its promoter, resulting in Wnt/β-catenin signaling activation and consequent promotion of stem-like phenotypes of colorectal cancer cells. Our present results suggest the essential role of IL-6/STAT3/miR-92a/Wnt/β-catenin pathway in regulating the stem cell-like traits of colorectal cancer cells and provide a potential target for colorectal cancer therapy.

## INTRODUCTION

Colorectal cancer (CRC) is one of the most lethal malignant tumors worldwide [1]. Despite advances in treatment modalities, the prognosis remains poor. The treatment failure may be the presence of cancer stem cells (CSCs) [2, 3]. CSCs have been defined as subpopulations of tumor cells exhibiting stem cell properties, which show the ability to self-renew and differentiation [4]. It is believed that CSCs mediate chemoresistance and subsequent tumor recurrence [5]. Therefore, therapies by suppressing CSCs function can likely improve the therapeutic effectiveness of CRC treatment.

Current findings support that Wnt/β-catenin pathway plays a crucial role in CSCs [6, 7]. A high level of Wnt activity in colon cancer cells stimulated by microenviornmental factors functionally designates the CSC population [8]. Inhibition of Wnt pathway has also been shown to be effective in counteracting stemness programs in CRC [9]. However, the mechanisms of Wnt/β-catenin pathway deregulation in CRC stem cell are not fully understood.

The canonical Wnt pathway results in β-catenin nuclear accumulation and transcriptional activation of target genes [10, 11]. The majority of colorectal cancers originate from benign adenomas, which are caused by inactivating mutations in the adenomatous polyposis coli (APC) gene. APC mutation leads to the constitutive activation of the Wnt/β-catenin pathway [12]. However, the mutation of APC cannot fully explain the reason of Wnt signaling induced CRC Stem Cell properties [8, 13, 14]. Thus, alternative mechanisms through which Wnt/β-catenin signaling were activated in CRC might exist.

MicroRNAs (miRNA) are endogenous small noncoding RNAs that can negatively regulate gene expression by directly binding to the 3’-untranslated region (3’-UTR) [15]. Distinct miRNAs have been found to directly regulate the Wnt/β-catenin pathway in human malignancies including CRC [16, 17]. Our previous study found that miR-92a induced epithelial-mesenchymal transition (EMT) and functioned as an oncogene in CRC cells [18, 19]. However, the regulatory effect of miR-92a on CRC stem cell and Wnt/β-catenin signaling currently remains unclear.

In this study, we reveal that miR-92a induces CRC cell chemoresistance and activates the stem cell-like properties. Moreover, we find that miR-92a, induced by its upstream activator IL-6/STAT3, can promote wnt/β-catenin signaling, and consequently enhance stem cell-like phenotypes of CRC.

## RESULTS

### MiR-92a is upregulated in chemoresistant CRC cells and tumor tissues

**Figure 1:**
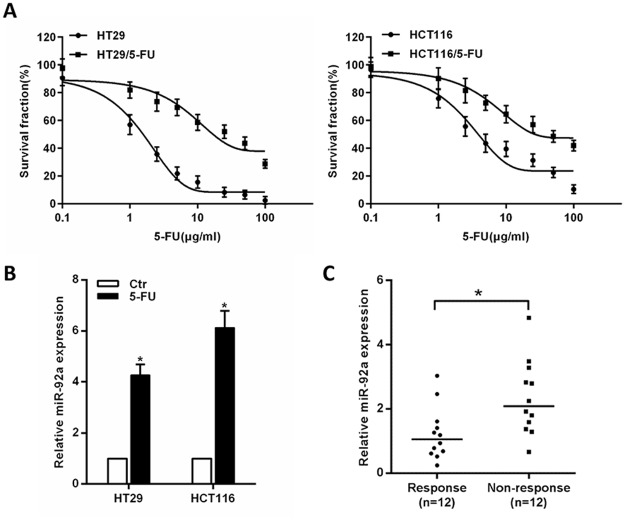
Expression of miR-92a correlates with CRC chemoresistance **(A)** Representative curves of growth inhibitory effects in 5-FU-resistant CRC cells (HT29/5-FU and HCT116/5-FU). **(B)** MiR-92a expression in 5-FU-resistant CRC cells. **(C)** MiR-92a expression in 12 responding and 12 non-responding colon tumors. ^*^P < 0.05.

To establish 5-FU-resistant CRC cell lines, HT-29 and HCT116 cells were treated with 5-FU and the resistant cells were selected. MTT assays indicated that HT29/5-FU (IC_50_: 23.31 μg/ml) and HCT116/5-FU (IC_50_: 43.72 μg/ml) cells displayed more resistant against 5-FU compared to the parental HT29 (IC_50_: 1.31 μg/ml) and HCT116 (IC_50_: 4.78 μg/ml) cells (Figure 1A). To better understand the relevance of miR-92a in drug resistance, we determined miR-92a expression levels in CRC cells. The results showed miR-92a was notably upregulated in 5-FU-resistant CRC cells (Figure 1B). Then, we analyzed miR-92a expression in human colon cancer specimens from 24 patients who had received neoadjuvant chemotherapy. Among them, 12 patients had responses, and 12 had no responses. As shown in Figure 1C, we found that miR-92a levels in non-responders were higher than that in responders. These data suggest that miR-92a plays an important role in drug resistance of CRC cancer.

### Overexpression of miR-92a induces chemoresistance

**Figure 2:**
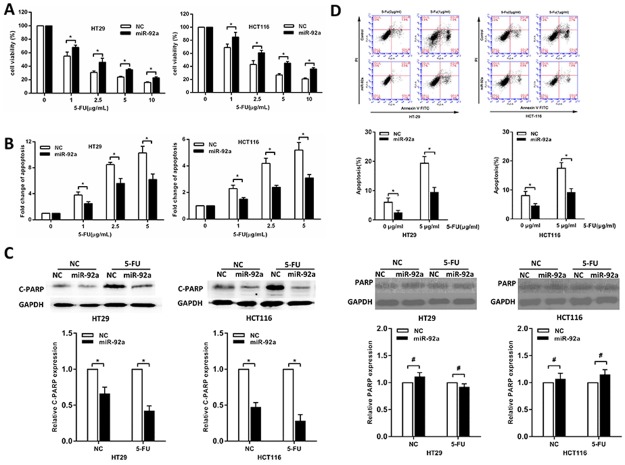
MiR-92a induces resistance to 5-FU treatment in CRC cells **(A, B)** MTT and DNA fragmentation assays were performed in miR-92a and control expressing cells treated with different concentrations of 5-FU for 72 h. **(C)** Western blot analysis of cleaved PARP (C-PARP) and PARP in miR-92a-expressing and control CRC cells after treatment with 10 μg/ml 5-FU for 72 h. **(D)** Flow cytometric analysis of miR-92a overexpression and control CRC cells treated with indicated concentrations of 5-FU. ^*^P < 0.05, ^#^not significance.

To clarify whether miR-92a affect the sensitivity of CRC cells to 5-FU, it was ectopically expressed in HT-29 and HCT116 cells. After exposure to 5-FU at different concentrations, miR-92a-expressing cells displayed increased cell viability (Figure 2A) and significantly reduced apoptosis compared with control cells (Figure 2B). Consistent with DNA fragmentation assays in Figure 2B, enforced expression of miR-92a resulted in a dramatic decrease of apoptosis as illustrated by western blot and flow cytometry (Figure 2C, 2D). These results indicated that miR-92a protected CRC cells from 5-FU-induced apoptosis. To further prove that miR-92a knockdown could restore 5-FU sensitivity, mice-bearing specific tumor xenografts were treated with 5-FU. As shown in Supplementary Figure 1, we found that antagomiR-92a significantly enhanced the sensitivity of CRC cells to 5-FU compared with controls. Therefore, these results confirm that miR-92a confers drug resistance in CRC cells.

### MiR-92a promotes sphere formation and expression of CSC markers

**Figure 3:**
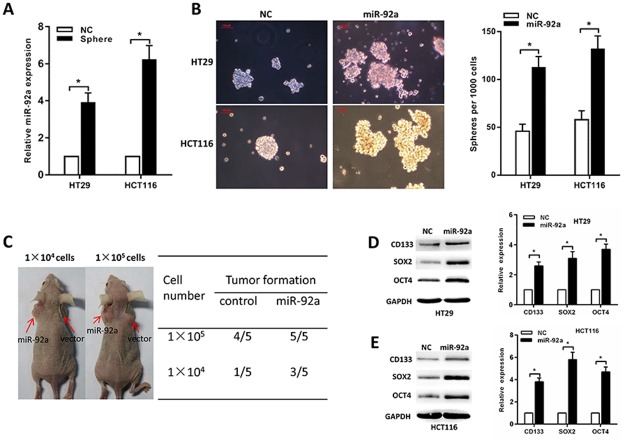
MiR-92a increases CRC stem cell-like properties **(A)** MiR-92a is highly expressed in HCT116 and HT29 spheres than normal HCT116 and HT29 cells. **(B)** Representative images showing sphere forming ability affected by miR-92a expression. Red scale bar represents 100 μm. **(C)** Tumor formation in nude mice shows increased *in vivo* tumorigenicity in HCT116/miR-92a cells compared with control cells. **(D, E)** Western blot and RT-PCR analyses of CD133, SOX2 and OCT4 expression in miR-92a or control cells. ^*^P < 0.05.

Because chemoresistance is a hallmark of CSCs, we determine whether miR-92a could enhance the stemness of CRC cells. First, we found that miR-92a is highly expressed in HCT116 and HT29 spheres than normal HCT116 and HT29 cells (Figure 3A). Second, a tumor sphere formation assay showed that miR-92a-transduced cells formed more and larger spheres, suggesting that miR-92a had self-renewal ability (Figure 3B). Tumor-initiating ability is another criterion for CSC properties, and we compared the tumor initiating ability of miR-92a-expressing cells with that of control cells. Following limiting dilution, we found that HCT116/miR-92a cells displayed a marked increase in tumorigenesis (Figure 3C). Finally, Western blot and RT-PCR analyses showed that HCT116/miR-92a and HT29/miR-92a spheres exhibited higher levels of CD133, SOX2 and OCT4 than normal HCT116 and HT29 spheres (Figure 3D, 3E). In summary, miR-92a reintroduction promotes the stem-like properties of CRC cells.

### MiR-92a activates Wnt/β-catenin signaling

**Figure 4:**
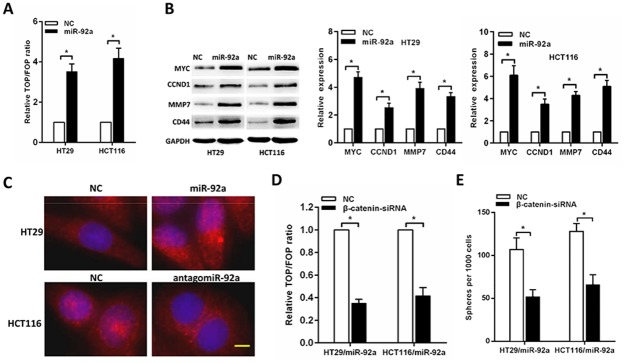
miR-92a activates Wnt/β-catenin signalling **(A)** TOP/FOP luciferase reporter assays in the indicated cells. **(B)** Western blot and RT-qPCR analyses of the expression of multiple Wnt/ β-catenin downstream genes in CRC cells. **(C)** Subcellular β-catenin localization in the indicated cells was assessed by immunofluorescence staining. Yellow scale bar represents 10 μm. **(D)** The stimulatory effect of miR-92a on TOP/FOP luciferase reporter activity was impaired by silencing β-catenin. **(E)** Sphere formation assays indicated that silencing β-catenin abrogated the promotive effects of miR-92a on self-renewal of CRC cells. ^*^P < 0.05.

Since the Wnt/β-catenin pathway contributes to the regulation of CSCs and has an important role in tumor progression of CRC, we then examined whether miR-92a has an effect on Wnt/β-catenin signalling. By using TCGA dataset and the GSEA assay, we found that the miR-92a expression was positively correlated with Wnt-activated gene signatures (Supplementary Figure 2). As expected, we found that miR-92a overexpression significantly increased luciferase activity of the TOP/FOP reporter (Figure 4A). In addition, Western blot and RT-PCR analyses of miR-92a-overexpressing CRC cells revealed that the expression levels of four wnt/β-catenin downstream genes were remarkably increased (Figure 4B). Meanwhile, immunofluorescence staining assay showed that miR-92a overexpression increased, whereas miR-92a inhibition decreased, the nuclear β-catenin accumulation in CRC cells (Figure 4C).

Next, we further examined the role of Wnt/β-catenin activation in miR-92a-induced stemness. As shown in Figure 4D, silencing β-catenin impaired the TOP/FOP luciferase reporter activity. Moreover, sphere formation assays indicated that silencing β-catenin abrogated the promotive effects of miR-92a on self-renewal of CRC cells (Figure 4E). These results demonstrate that wnt/β-catenin signaling is essential for miR-92a-induced stem-like traits in CRC.

### MiR-92a activates wnt/β-catenin signaling by targeting DKK3, KLF4 and GSK3β

**Figure 5:**
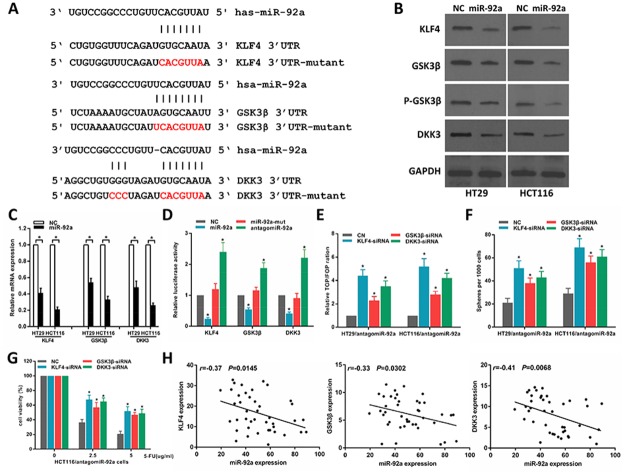
MiR-92a directly targets multiple negative regulators of wnt/β-catenin pathway **(A)** Predicted binding sites of miR-92a in the wild type 3’-UTRs of KLF4, GSK3β and DKK3. Mutations in these 3’-UTRs are highlighted in red. **(B, C)** Western blot and RT–qPCR analyses of KLF4, GSK3β and DKK3 expression in the indicated cells. **(D)** Luciferase activity of targets 3’UTR in HCT116 cells. **(E)** The inhibitory effect of antagomiR-92a on TOP/FOP luciferase reporter activity was impaired by silencing KLF4, GSK3β and DKK3. **(F)** Sphere formation assays indicated that silencing KLF4, GSK3β and DKK3 abrogated the suppressive effects of antagomiR-92a on self-renewal of CRC cells. **(G)** MTT assays indicated that silencing KLF4, GSK3β and DKK3 abrogated antagomiR-92a-induced sensitivity to 5-FU in CRC cells. **(H)** Correlation of miR-92a with KLF4, GSK3β and DKK3 expression in 42 clinical CRC specimens. ^*^P < 0.05.

To investigate the mechanism of miR-92a on Wnt/β-catenin signalling activation in CRC cells, we first sought to identify potential miR-92a target genes using publicly algorithms (TargetScan and miRanda). We identified three tumor suppressor genes associated with Wnt pathway, namely, DKK3, KLF4 and GSK3β, that might be the potential targets of miR-92a (Figure 5A). Western blot and RT–PCR analysis revealed that miR-92a overexpression repressed KLF4, GSK3β and DKK3 expression levels (Figure 5B, 5C). Luciferase assays showed that miR-92a associated directly with the 3’UTRs of KLF4, GSK3β and DKK3 (Figure 5D). We then examined the role of KLF4, GSK3β and DKK3 in miR-92a- induced stemness. As shown in Figure 5E-5G, silencing of KLF4, GSK3β and DKK3 could rescue the TOP/FOP luciferase reporter activity, self-renewal ability and antagomiR-92a-elicited chemosensitivity of miR-92a-inhibited CRC cells. Moreover, a negative correlation between miR-92a and KLF4, GSK3β and DKK3 expression was found in CRC specimens (Figure 5H). Our results reveal that DKK3, KLF4 and GSK3β are the key regulators for miR-92a-induced wnt/β-catenin activation and stem cell-like phenotype in CRC cells.

### MiR-92a is upregulated by IL-6/Stat3 signaling

**Figure 6:**
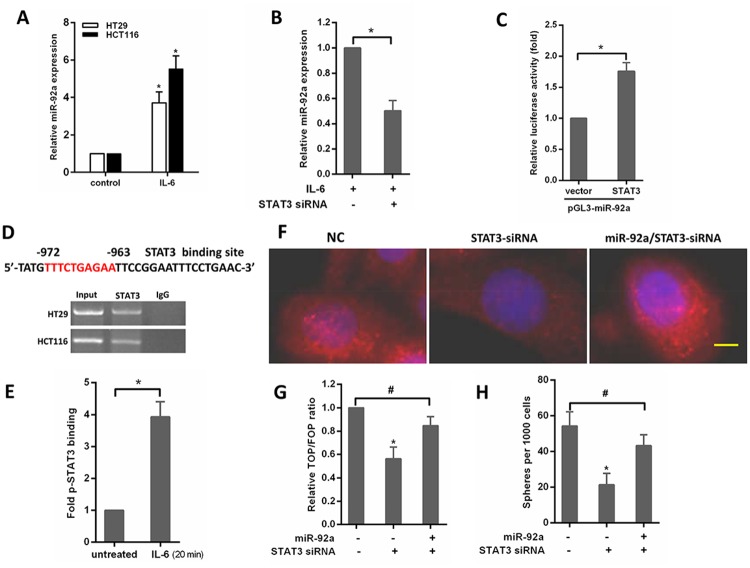
IL-6/STAT3 upregulates miR-92a expression to induce stemness of CRC cells **(A)** Expression of miR-92a in CRC cells after treatment with 20ng/mL IL-6 for 48 hours. **(B)** qPCR analysis of miR-92a in HT29 cells transfected with STAT3 siRNAs for 24 hours and subsequently treated with IL-6 for 48 hours. **(C)** Analysis of the luciferase activity of pGL3-miR-92a promoter report plasmid cotransfected with an empty vector or STAT3 plasmid in HT29 cells. **(D)** Top, schematic diagram indicates the location and sequence of putative STAT3-binding site on miR-92a promoter region. Bottom, ChIP assays were performed in HT29 and HCT116 cells. **(E)** ChIP analysis for p-STAT3 bound to miR-92a promoter in untreated or IL-6–treated HCT116 cells. **(F)** immunofluorescence images of nclear translocation of β-catenin in HCT116 cells. Yellow scale bar represents 10 μm. **(G)** Assay of TOP/FOP luciferase activity in HCT116 cells transduced with STAT3-siRNA or miR-92a/STAT3-siRNA. **(H)** The effects of STAT3- siRNA or miR-92a/STAT3-siRNA on sphere formation in HCT116 cells. ^*^P < 0.05, ^#^not significance.

It is known that STAT3 transcriptionally upregulates miR-92a in cancer cells [20], and the promoter region of the miR-17-92 cluster genes contains a conserved binding site for STAT3 [21]. Therefore, we speculated activation of IL-6/STAT3 signaling might be a potential mechanism for miR-92a up-regulation during colorectal carcinogenesis. Indeed, the expression of miR-92a increased after exposure to IL-6 in CRC cells (Figure 6A). IL-6-induced up-regulation of the miR-92a was abolished by siRNA knockdown of STAT3 (Figure 6B). To confirm the transcriptional control of miR-92a by STAT3, we constructed reporter plasmids containing the miR-92a promoter. The results showed that ectopic expression of STAT3 upregulated miR-92a promoter activity in HT29 cells (Figure 6C). Additionally, ChIP assay confirmed the enrichment of STAT3 on the promoter region of miR-92a (Figure 6D). Moreover, IL-6 treatment increased the association of phosphorylated STAT3 (p-STAT3) to its DNA binding site in miR-92a promoter (Figure 6E). We also examined whether miR-92a affects the function of STAT3 in the stemness and wnt/β-catenin signaling regulation of CRC cells. As shown in Figure 6F, knockdown of STAT3 suppressed Wnt/β-catenin signaling by downregulation of nuclear translocation of β-catenin, decreased TOP/FOP luciferase reporter activity (Figure 6G) and sphere-forming capacity in CRC cells (Figure 6H), while these suppressive effects could be rescued by miR-92a. These findings suggest that IL-6/STAT3 pathway increases miR-92a expression by directly targeting the miR-92a promoter, resulting in Wnt/β-catenin signaling activation and consequent promotion of stemness of CRC cells.

### MiR-92a expression is clinically correlated with STAT3 and β-catenin

**Figure 7:**
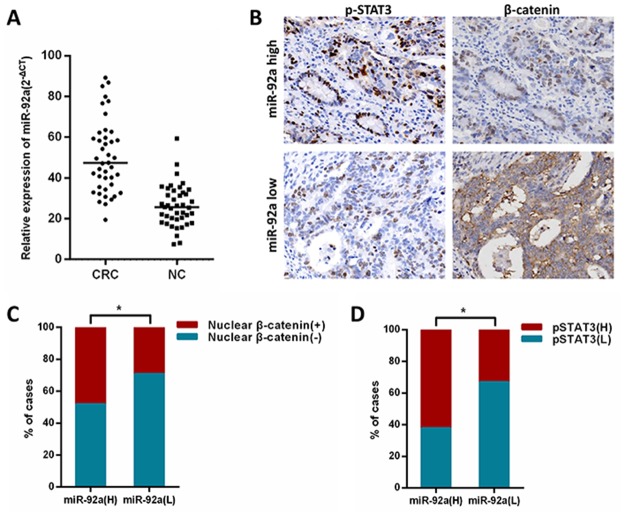
Clinical relevance of miR-92a with STAT3 and β-catenin in human CRC tissues **(A)** Expression ofmiR-92a in primary CRC tissues and matched noncancerous tissues(NC) were analyzed by qRT-PCR. **(B)** immunohistochemical analysis of β-catenin and p-STAT3 expression in serial sections of CRC specimens.×400. **(C, D)** Levels of miR-92a expression were positively correlated with expression of nuclear β-catenin and pSTAT3 in CRC tissues. ^*^P < 0.05.

Finally, to test whether the regulation described above in CRC tissues is clinically relevant, 42 clinical CRC specimens were divided into high or low miR-92a subgroups in relation to median value (Figure 7A). As shown in Figure 7B, we found that high miR-92a levels tended to increase the expression of nuclear β-catenin and nuclear pSTAT3. The 42 CRC cancer patient cases were then divided into groups with relatively levels of nuclear β-catenin and pSTAT3. From this analysis, we observed that miR-92a expression was shown to be positively correlated with nuclear β-catenin and nuclear pSTAT3 expression (Figure 7C, 7D), further supporting the notion that miR-92a is upregulated by IL/STAT3 and activates Wnt/β-catenin pathway in CRC.

## DISCUSSION

MiRNAs have been observed in various types of cancer and may be involved in modulating cancer cell behaviors [22, 23]. MiR-92a plays an important role in several malignant tumors [24, 25]. We previously reported that miR-92a induced EMT in CRC [18]. The EMT process has been reported to be associated with cancer stem cell properties [26]. MiR-92a is also found to regulate the stem cell-like properties of glioma and gastric cancer, suggesting that miR-92a might also be involved in CRC stemness [27, 28].

In this study, we found that mR-92a was upregulated in chemoresistant CRC tissues and cells. Ectopic expression of miR-92a conferred resistance to 5-FU-induced apoptosis *in vitro*, while antagomiR-92a significantly enhanced the drug sensitivity of CRC cells *in vivo*. These findings establish a novel role of miR-92a in supporting the development of chemoresistance, and provide a molecular basis for targeting miR-92a or its downstream effectors to overcome chemo- resistance in CRC.

The existence of CRC stem cells is considered to be the origin of chemoresistance and CRC recurrence. Consistently, we found miR-92a is highly expressed in CRC cell spheres, and overexpressing miR-92a promotes sphere formation and expression of CSC markers in CRC cells. MiR-92a overexpression also promoted tumorigenesis *in vivo*. These results demonstrate miR-92a may play an important role in CRC stem cells.

The Wnt/β-catenin signalling pathway is a well-known pathway involved in regulating CSCs in gastrointestinal tract [29]. In the present study, we found miR-92a activates Wnt/β-catenin pathway through directly downregulation of KLF4, GSK3β, and DKK3. KLF4, GSK3β, and DKK3 are negative regulators of wnt/β-catenin signaling and involved in CRC stem cell properties [30, 31]. KLF4, a member of the Krüppel-like transcription factor family, suppressed Wnt/β-catenin pathway by inhibiting the β-catenin transactivating domain in CRC cells [32, 33]. GSK3β is a negative regulator of canonical Wnt signaling and downregulated in HCT116 and HT29 spheroid cells [34, 35]. In this study, we further confirmed that miR-92a promoted CRC sphere formation by directly targeting GSK3β. It is also known that Akt activation phosphorylated GSK3β at Ser9 and inhibited GSK-3β activity, leading to nuclear β-catenin accumulation and ultimately triggering tumor progression [36, 37]. In CRC cells, miR-92a was reported to promote metastasis via suppression of PTEN expression and activation of the PI3K/AKT pathway [38]. We therefore think miR-92a might regulate PTEN/AKT/GSK-3β signaling, thus affecting the Wnt/β-catenin pathway in CRC cells. DKK3, another Wnt suppressor, can inhibite β-catenin nuclear translocation in CRC cells [39]. Our present study showed that miR-92a activated Wnt/β-catenin signalling and promoted CRC stem-like properties and tumorigenesis.

STAT3, a transcription factor that is constitutively activated in several cancer types, is considered to be an oncogene [40]. A major driver of STAT3 activation in malignancy is the IL-6, which signals through a complex of IL-6 receptor and gp130 to activate JAKs and induce tyrosine phosphorylation of STAT3 [41]. Previous studies have indicated that STAT3 is necessary for the maintenance of colon cancer–initiating cells [42]. MiR-92a is a member of the miR-17–92 cluster [43]. A recent study showed miR-17–92 was regulated by IL-6/STAT3 in cholangiocarcinoma cells [44]. We reported here that IL-6/Stat3 signaling transcriptionally regulated the expression of the miR-92a in CRC cells. Moreover, IL-6/Stat3 can regulate sphere-forming capacity and Wnt/β-catenin signaling by targeting miR-92a. In this context, it is notable that recent studies have shown that miR-92a targets JAK-STAT pathway inhibitor SOCS5 [45]. Thus, it is possible that the IL-6/STAT3 induced miR-92a may further amplify IL-6/Stat3 signaling through targeting SOCS5, forming a positive feed-forward loop that drives CRC stem-like properties and carcinogenesis.

In summary, our work provides evidence that IL-6/STAT3 pathway increases miR-92a expression by directly targeting the miR-92a promoter, resulting in Wnt/β-catenin signaling activation by targeting KLF4, GSK3β, and DKK3, and consequently promoting stem-like phenotypes of CRC cells. The newly identified IL-6/STAT3/miR-92a/Wnt axis helps to further elucidate the complex molecular mechanisms which regulate CRC stem cell-like properties, and represents a novel strategy for the treatment of patients with CRC.

## MATERIALS AND METHODS

### Tissue specimens

24 tumor tissues from patients with locally advanced colon cancer received neoadjuvant chemotherapy (5-FU-based chemotherapy) prior to surgical removal of the tumors at the Department of Gastrointestinal Surgery, The Affiliated Hospital of North Sichuan Medical College. Response was assessed by computer-assisted tomography in lymph node, liver and lung metastases, as well as in primary lesions. To be classified as a responder, a tumor had to have a 50% reduction in the sum of the diameters of the indicator lesion without growth of other disease or the appearance of new lesions [46]. To test the clinical relationship of miR-92a with STAT3 and β-catenin, we recruited a second cohort comprising 42 tumor samples. All tissue samples were immediately frozen in liquid nitrogen and stored at -80 C until the extraction of RNA. The specimens were stained with hematoxylin and eosin and examined histopathologically. Sections that consisted of >80% carcinoma cells were used to prepare the total RNA. Corresponding formalin fixed and paraffin embedded tissues were available from 42 samples. Informed written consent was obtained from each patient, and research protocols were approved by the Medical Ethics Committee of North Sichuan Medical College.

### Cell culture

The human CRC cell lines HT29 and HCT116 were obtained from the American Type Culture Collection. The cell lines were cultured in DMEM (Invitrogen, Carlsbad, CA, USA) supplemented with 10 % fetal bovine serum (FBS; Invitrogen). 5-FU-resistant CRC cell lines (HT29/5-FU and HCT116/5-FU) was generated by incubating HT29 and HCT116 cells with increasing level of 5-FU until the concentration reached 2 μg/ml. Resistant cell lines were maintained under constant treatment with drug for daily culture.

### RNA extraction and quantitative real-time PCR

Total RNA, including miRNA, was isolated from tissues or cell lines using TRIzol reagent (Invitrogen) according to manufacturer's instructions. For miRNA expression analysis, reverse transcription was performed using the TaqMan microRNA reverse transcription kit (Applied Biosystems, Foster City, CA, USA) with miR-92a specific primers (Applied Biosystems). Mature miR-92a levels were quantified with TaqMan miRNA assays (Applied Biosystems). For DKK3, KLF4, GSK3β, CD133, SOX2 and Oct4 mRNA detection, reverse transcription was performed using the PrimeScript RT reagent Kit (Takara, Dalian, China). Quantitative PCR was performed using SYBR Premix Ex Taq (Takara) on the ABI 7500 real-time PCR System (Applied Biosystems). U6 snRNA or β-actin was used as internal control. The primer Sequences are listed in Supplementary Table 1. The relative expression levels were calculated by the equation 2^-ΔΔCT^.

### Cell viability and apoptosis assays

CRC cells were plated in 96-well plates at 5×10^3^ per well in a final volume of 100 μl, and 20 μl of 5 mg/ml MTT was added to each well at the indicated time. After incubation at 37 °C for 4 h, the MTT solution was removed, and 150 μl dimethyl sulfoxide (DMSO) was added to each well followed by measuring the absorbance at 570 nm on a SpectraMax M5 microplate reader (Molecular Devices, Sunnyvale, CA, USA). Cell viability was calculated as the ratio of the absorbance values of drug-treated samples to those of controls. Apoptosis was detected using a DNA fragmentation ELISA kit (Roche Applied Science, USA).

### Sphere formation assay

In total, 1×10^3^ cells were seeded in 6-well ultra-low cluster plates (Corning Inc., Corning, NY) for 10 days. Spheres were cultured in DMEM/F12 serum-free medium (Invitrogen) supplemented with 2% B27 (Invitrogen), 20 ng/ml EGF, 20 ng/ml bFGF, 0.4% BSA, and 5 μg/ml insulin (Sigma).

### Immunohistochemistry and immunofluorescence

For immunohistochemistry, sections were incubated with antibody against p-STAT3 (Tyr 705, Cell Signaling Technology, Danvers, MA) and β-catenin (Cell Signaling Technology), followed by incubation with secondary antibodies. Expression levels were visualized and classified as previously described [47]. For immunofluorescence, cells were grown on glass culture slides (BD Biosciences) and fixed with 4% cold methanol at -20°C for 10 min. Subsequently, cells were blocked with 10% goat serum for 1 h and incubated with primary antibodies against β-catenin (Cell Signaling Technology) at 4 °C for 1 h and then incubated with florescent labeled secondary antibodies for 1h at room temperature. After counterstained with DAPI (Invitrogen), the slide was observed under a confocal microscope (Zeiss).

### Western blot analysis

Total proteins were prepared from fresh-frozen tissue or cultured cell samples by complete cell lysis (Roche) with protease and phosphatase inhibitors. After gel transference to PVDF (Millipore, Bedford, MA), the antibodies KLF4, CD44, CCND1 (Abcam, Cambridge, MA), OCT4, MMP-9, SOX2, CD133, c-MYC, GSK3β, p-GSK3β (Ser9), DKK3, PARP and Cleaved PARP (Cell Signaling Technology) were used. Subsequently, suitable secondary antibodies were applied and bands for specific molecules were detected by enhanced chemiluminescence (ECL; Millipore). Each sample was normalized to GAPDH (Santa Cruz Biotechnology, Santa Cruz, CA, USA).

### Plasmids and transfection

The sequence of pre-miR-92a was cloned into the pMSCV-puro plasmid. The pMSCV-miR-92a or empty pMSCV vector were co-transfected into HEK293T cells with the retroviral packaging PIK vector using the calcium phosphate method. After transfection, the cell supernatants were collected and used to infect HCT116 cells, and the stably transfected cells were selected using puromycin and confirmed by quantitative RT-PCR. TOP/FOP reporter plasmids were purchased from Upstate Biotechnology (Lake Placid, NY, USA). Antagomir-92a and corresponding control oligonucleotide were purchased from RiboBio (Guangzhou, China). Small interfering RNA (siRNA) against STAT3, β-catenin, DKK3, KLF4 and GSK3β were purchased from Genechem (Shanghai, China). The coding sequences of STAT3 that was amplified by PCR and subcloned into vector pcDNA 3.1(Invitrogen) using the primers listed in Supplementary Table 1. The empty vector served as a negative control. Transfection of plasmids or oligonucleotides was performed using Lipofectamine 2000 reagent (Invitrogen) according to the manufacturer's instructions.

### Flow cytometric analysis

CRC cells were placed in 6-well plates at concentrations of 2×10^5^ per well. After 24h of incubation, the cells were exposed to 0 or 1μg/ml 5-FU and cultured for 24h. Cell apoptosis was detected using Annexin V-FITC/PI apoptosis detection kit (KeyGEN BioTECH, China) and analyzed by FACS (BD Accuri C6, USA) according to the manufacturer's instructions.

### *In vivo* animal study

Six-week-old female BALB/c nude mice were obtained from Shanghai Laboratory Animal Center (Shanghai, China). All animal studies were conducted according to protocols approved by the Committee on the Ethics of Experimental Animal of North Sichuan Medical College. For subcutaneous tumor models, 2 × 10^6^ control or antagomiR-92a cells were injected into the flanks of nude mice (n=5). One week after injection, 5-FU (40 mg/kg/day) or control was administered by intraperitoneal injection for 5 consecutive days /week for 2 weeks [48]. Four weeks after injection, the mice were killed and the end-point tumor mass were weighted. For the limiting dilution assay, CRC stem-like cells (1×10^4^ and 1×10^5^) transfected with miR-92a or control were injected subcutaneously into bilateral flanks of mice (n=5). Engrafted mice were inspected weekly for tumor development by visual observation and palpation until 8 weeks postinjection.

### Luciferase reporter assay

To validate whether KLF4, DKK3 and GSK3β are direct targets of miR-92a, wild-type or mutant 3′-UTR of KLF4, DKK3 and GSK3β were cloned into the psicheck-2 vector (Promega, Madison, WI, USA). HCT116 cells were co-transfected with miR-92a or antagomiR-92a and wild-type or mutant 3′-UTR-luc by using Lipofectamine 2000. To validate the STAT3-binding sites in the miR-92a promoter, the miR-92a promoter was amplified from human genomic DNA to generate miR-92a promoter using specific primers. The PCR product was cloned into the pGL3-basic vector (Promega). HT29 cells were transfected with pGL3-miR-92a along with pcDNA3.1-STAT3 expression vector or an empty vector using Lipofectamine 2000. After transfection for 48h, cells were harvested and assayed with Dual-Luciferase Reporter Assay System (Promega) according to the manufacturer's protocols. The primers used in aforementioned construction or mutation are listed in Supplementary Table 1.

### Chromatin immunoprecipitation assay (ChIP)

ChIP assay was performed using EZ ChIP Assay Kit (Millipore) following the manufacturer's instructions. Briefly, cells were fixed in 1% formaldehyde to cross-link DNA and proteins. Then, the cells were lysed with protease and phosphatase inhibitors. DNA was immunoprecipitated by p-STAT3 (Tyr 705, cell signaling technology). Normal Rabbit IgG was used as negative control. The samples were then reversed and purified. The immunoprecipitated DNA was amplified by qPCR for specific sequences containing putative STAT3 binding sites. Primers are listed in Supplementary Table 1.

### Bioinformatics analysis

The putative promoter sequence of miR-92a was retrieved from UCSC Genome Brower (http://www.genome.ucsc.edu/). Prediction of transcription factors for miR-92a was conducted using (http://jaspar.genereg.net/). Gene expression profiles of Colon adenocarcinoma were downloaded from The Cancer Genome Atlas (TCGA) database (https://tcga-data.nci.nih.gov/tcga/). The association between gene expression and biological processes was analyzed using Gene Set Enrichment Analysis (GSEA) (http://www.broadinstitute.org/gsea/index.jsp).

### Statistical analysis

All statistical analyses were carried out using SPSS statistical software (Version 19, IBM SPSS, Chicago, IL, USA). Statistical significance between groups was analyzed by Student's t-test, while categorical data were studied using chi-square test. Bivariate correlations between study variables were calculated by Pearson correlation coefficients. Statistical significance was defined as *P* < 0.05.

## SUPPLEMENTARY MATERIALS FIGURES AND TABLE



## Abbreviations

CRC: colorectal cancer
5-Fu: 5-fluorouracil
miR: microRNAs
CSC: cancer stem cells
3’-UTR: 3’-untranslated regions
GAPDH: glyceraldehyde-3-phosphate dehydrogenase
PBS: phosphate-buffered saline
RT-PCR: reverse transcription-polymerase chain reaction
STAT3: signal transducer and activators of transcription 3
EMT: epithelial-mesenchymal transition
