# Supplementary material for: MiR-92a promotes stem cell-like properties by activating Wnt/β-catenin signaling in colorectal cancer
Source: Oncotarget. 2017 Oct 9;8(60):101760–70. doi: 10.18632/oncotarget.21667 (PMC5731912; doi:10.18632/oncotarget.21667)
Supplement: Supplementary file 1 [file oncotarget-08-101760-s001.pdf]

MiR-92a promotes stem cell-like properties by activating Wnt/β-catenin signaling in colorectal cancer

SUPPLEMENTARY MATERIALS

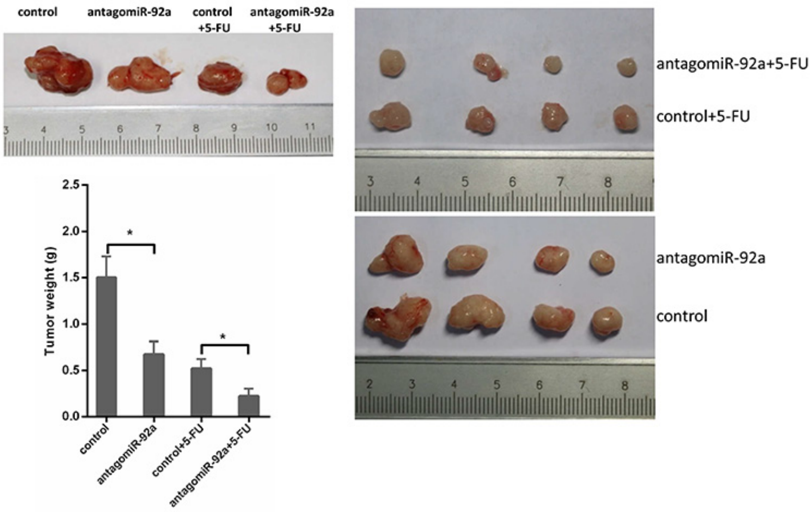

Supplementary Figure 1: Subcutaneous tumor model was used to assess the effect of antagomiR-92a on the chemoresistance of HCT116 cells *in vivo*.

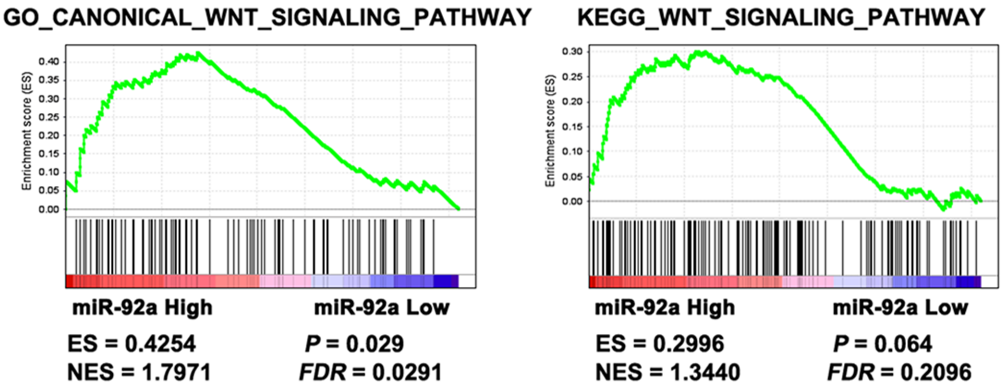

Supplementary Figure 2: GSEA plot showing that miR-92a expression was positively correlated with Wnt-activated gene signatures.

Supplementary Table 1: Primer sequences used for PCR amplification

**Primers for real-time quantitative RT-PCR**		
KLF4	5′-CGAACCCACACAGGTGAGAA-3′	5′-TACGGTAGTGCCTGGTCAGTTC-3′
DKK3	5′-TCACATCTGTGGGAGACGAA-3′	5′-CAGGTGTACTGGAAGCTGGC-3′
CD44	5′-GACAAGTTTTGGTGGCACG-3′	5′-CACGTGGAATACACCTGCAA-3′
MYC	5′-CACCGAGTCGTAGTCGAGGT-3′	5′-GCTGCTTAGACGCTGGATTT-3′
GSK3β	5′-CCACTGTTGTCACCTTGCTG-3′	5′-GAGTGATCATGTCAGGGCG-3′
CCND1	5′-AGAGGCGGAGGAGAACAAAC-3′	5′-GGCGGATTGGAAATGAACTT-3′
MMP7	5′-GCATCTCCTTGAGTTTGGCT-3′	5′-GAGCTACAGTGGGAACAGGC-3′
GAPDH	5′-GACTCATGACCACAGTCCATGC-3′	5′-AGAGGCAGGGATGATGTTCTG-3′
**Primer Sequences used for PCR amplification of plasmid construction**		
STAT3	5′-GCTAAGCTTTATGGCCCAATGGAATCA GCTACAG-3′(HindIII)	5′-GCTCTCGAGTCATGGGGGAGGTAGCG CACTCCG-3′(XhoI)
**Primers for luciferase plasmids construction**		
miR-92a-pGL3	5′-CACAACTCGAGAGGAAGTATGAGCGA AACCCT-3′ (XhoI)	5′-CAAAAGCTTCGCTTTACTACGACCGG AGG-3′ (HindIII)
**Primers for gene 3′UTR luciferase plasmid construction**		
KLF4 wt	5′-CACAACTCGAGCCCAGCCAGAAAGCA CTACA-3′ (XhoI)	5′-AAGGAAAAAAGCGGCCGCTCTGCTTA AGGCATACTTGGGA-3′ (NotI)
KLF4 mut	5′-TGCATTTCCCTACTTGGTCTGGCTTTAA GAATGTCCTCTTGG-3′ (XhoI)	5′-AAGTTCCCTTTTGCAATAGTAATCCAA ACCAAAAAGCTCTGAGC-3′ (NotI)
DKK3 wt	5′-CACAACTCGAGATCTGGACCAGGCTGT GGG-3′ (XhoI)	5′-CACAACACAAGCGGCCGCGATGTTTG CAAAGCAGGGCA-3′ (NotI)
DKK3 mut	5′-CACAACTCGAGATCTGGACCAGGCTGT CCCTAGATCACGTTAAGAAATAGCTAATT TA-3′ (XhoI)	5′-CACAACACAAGCGGCCGCGATGTTTG CAAAGCAGGGCA-3′ (NotI)
GSK3β wt	5′-CACAACTCGAGAACAGCAGATTCTGG GAGCC-3′ (XhoI)	5′-CACAACACAAGCGGCCGCAGTGATCC ATCGGCAACACA-3′ (NotI)
GSK3β mut	5′-GCTATTCACGTTATACTAGCAGTTACTG CACGGAG-3′ (XhoI)	5′-CTAGTATAACGTGAATAGCATTTTAGA CCACTGAC-3′ (NotI)
**Primers for ChIP assay promoter-specific PCR**		
miR-92a	5′-TGTACAATCACGAACCAGTGA-3′	5′-AGCATAGCTCGGAAAGAACCTC-3′
